# Extended-release calcifediol in stage 3–4 chronic kidney disease: a new therapy for the treatment of secondary hyperparathyroidism associated with hypovitaminosis D

**DOI:** 10.1007/s40620-021-01152-5

**Published:** 2021-10-09

**Authors:** Mario Cozzolino, Paola Minghetti, Pierluigi Navarra

**Affiliations:** 1 Renal Division, ASST Santi Paolo e Carlo, Department of Health Sciences, University of Milan, Milan, Italy; 2 Department of Pharmaceutical Science, Università degli Studi di Milano, Milan, Italy; 3 Department of Healthcare Surveillance and Bioethics, Section of Pharmacology, Catholic University Medical School, Fondazione Policlinico Universitario A. Gemelli-IRCCS, Rome, Italy

**Keywords:** Chronic kidney disease, Calcifediol, Parathyroid hormone, Secondary hyperparathyroidism, Vitamin D, Vitamin D insufficiency

## Abstract

A high percentage of patients with chronic kidney disease have hypovitaminosis D, which is a driver of secondary hyperparathyroidism and an important factor in chronic kidney disease-mineral and bone disorder. Vitamin D deficiency (serum total 25-OH vitamin D levels < 30 ng/mL) occurs early in the course of chronic kidney disease and treatment guidelines recommend early intervention to restore 25-OH vitamin D levels as a first step to prevent/delay the onset/progression of secondary hyperparathyroidism. The vitamin D forms administered to replace 25-OH vitamin D include cholecalciferol, ergocalciferol, and immediate- or extended-release formulations of calcifediol. Most patients with intermediate-stage chronic kidney disease will develop secondary hyperparathyroidism before dialysis is required. Control of parathyroid hormone levels becomes a major focus of therapy in these patients. This article focuses on the position of extended-release calcifediol in the treatment of patients with stage 3–4 chronic kidney disease and secondary hyperparathyroidism with hypovitaminosis D. Several characteristics of extended-release calcifediol support its use in the intermediate stages of chronic kidney disease. The pharmacokinetics of extended-release calcifediol make it effective for replenishing 25-OH vitamin D levels, with minimal impact on vitamin D catabolism from fibroblast-growth factor-23 and CYP24A1 upregulation. Extended-release calcifediol increases circulating 25-OH vitamin D levels in a dose-dependent manner and lowers parathyroid hormone levels by a clinically relevant extent, comparable to what can be achieved by administering active vitamin D analogues, though with a lower risk of hypercalcaemia and hyperphosphataemia. Active vitamin D analogues are reserved for patients undergoing dialysis or pre-dialysis patients with severe progressive secondary hyperparathyroidism.

**Graphic abstract:**

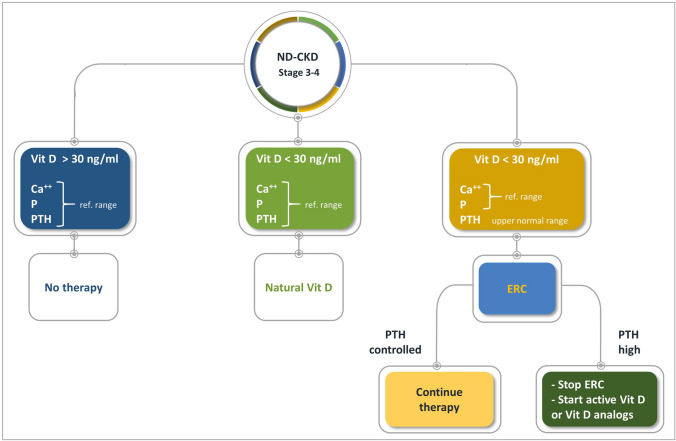

## Introduction

Chronic kidney disease (CKD) is a major public health problem, with an estimated global prevalence of 11–13% [1]. CKD is progressive, with most patients developing CKD-mineral and bone disorder and increased risks of fractures, cardiovascular disease, and reduced life expectancy [2–4]. CKD disrupts phosphorus and calcium homeostasis, and these perturbations lead to secondary hyperparathyroidism characterized by elevated parathyroid hormone levels and parathyroid hyperplasia [5], which are early events in most patients [6]. Secondary hyperparathyroidism is a critical component of CKD-mineral and bone disorder that manifests early during the progression of renal disease, occurring in approximately 40 to 80% of patients with stage 3 or 4 CKD [6]. In patients with stage 3/4 CKD, parathyroid hormone levels independently predict fractures, vascular events and death [7].

Phosphate retention and accumulation due to kidney damage is an early driver of secondary hyperparathyroidism. High serum phosphate triggers the release of fibroblast growth hormone 23 (FGF23) from bone, which, in turn, increases parathyroid hormone levels and inhibits activation of 25-hydroxy vitamin D (25(OH)D) by suppressing 25(OH)D-1α-hydroxylase expression [8]. Low levels of activated 1,25(OH)2D promote the progression of secondary hyperparathyroidism via multiple pathways, including decreased intestinal absorption of calcium that further stimulates parathyroid hormone secretion [6]. This causes progressive parathyroid gland hyperplasia with associated reduced responsiveness to calcium, vitamin D and FGF23 levels that gradually leads to autonomous parathyroid hormone secretion, (tertiary hyperparathyroidism) [9]. Early intervention is needed to slow progression, preserve bone health and minimise ectopic calcification.

The prevalence of vitamin D insufficiency is higher in patients with CKD than in the general population [6, 10, 11]. Causes may include decreased vitamin D activation and increased catabolism due to the effects of elevated FGF23 expression [12, 13]. Although there is no consensus regarding optimal 25(OH)D levels in patients with CKD [14], guidelines define vitamin D sufficiency as serum total 25(OH)D levels ≥ 30 ng/mL [15], which is consistent with the 2011 Endocrine Society definition of 30–100 ng/mL [16]. Studies in patients with CKD suggest that higher 25(OH)D levels are required to suppress parathyroid hormone levels and effectively treat secondary hyperparathyroidism [17–19].

Current guidelines recommend monitoring vitamin D status in pre-dialysis patients with CKD and supplementing with nutritional vitamin D (cholecalciferol or ergocalciferol) to delay the onset of secondary hyperparathyroidism [15, 20].

This article focuses on the position of Rayaldee^®^, an extended-release (in Europe the term “prolonged-release” is used) calcifediol formulation that raises 25(OH)D, lowers parathyroid hormone levels, and is approved for the treatment of secondary hyperparathyroidism in adults with CKD Stage 3 or 4 and vitamin D insufficiency or deficiency [21, 22].

## Issues with current management of secondary hyperparathyroidism in pre-dialysis CKD

Currently, the best strategy for 25(OH)D administration aimed at treating secondary hyperparathyroidism in patients with CKD remains controversial [23]. The choice is complicated and CKD stage-dependent. Several treatment options exist (Table 1). The ideal agent would normalize 25(OH)D levels and suppress parathyroid hormone, without significantly affecting FGF23 levels or calcium and phosphorus homeostasis. Such an agent should have a low risk for hypercalcaemia, ectopic calcification and oversuppression of parathyroid hormone.

**Table 1:**
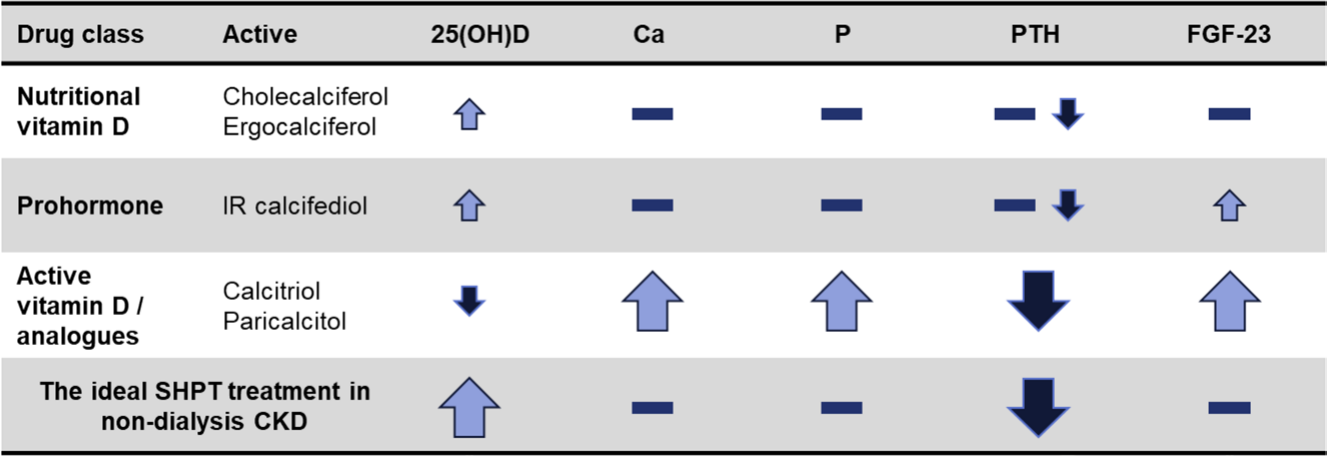
The effect of current treatment options for secondary hyperparathyroidism in patients with stage 3-4 CKD on mineral and bone disorder parameters (Adapted from Sprague et al. [24])

*Ca* calcium, *CKD* chronic kidney disease, *FGF-23* fibroblast growth factor 23, *IR* immediate release, *MBD* mineral and bone disorder, *P* phosphate, *PTH* parathyroid hormone, *secondary hyperparathyroidism* secondary hyperparathyroidism, *25(OH)D* 25-hydroxyvitamin D

**Fig. 1:**
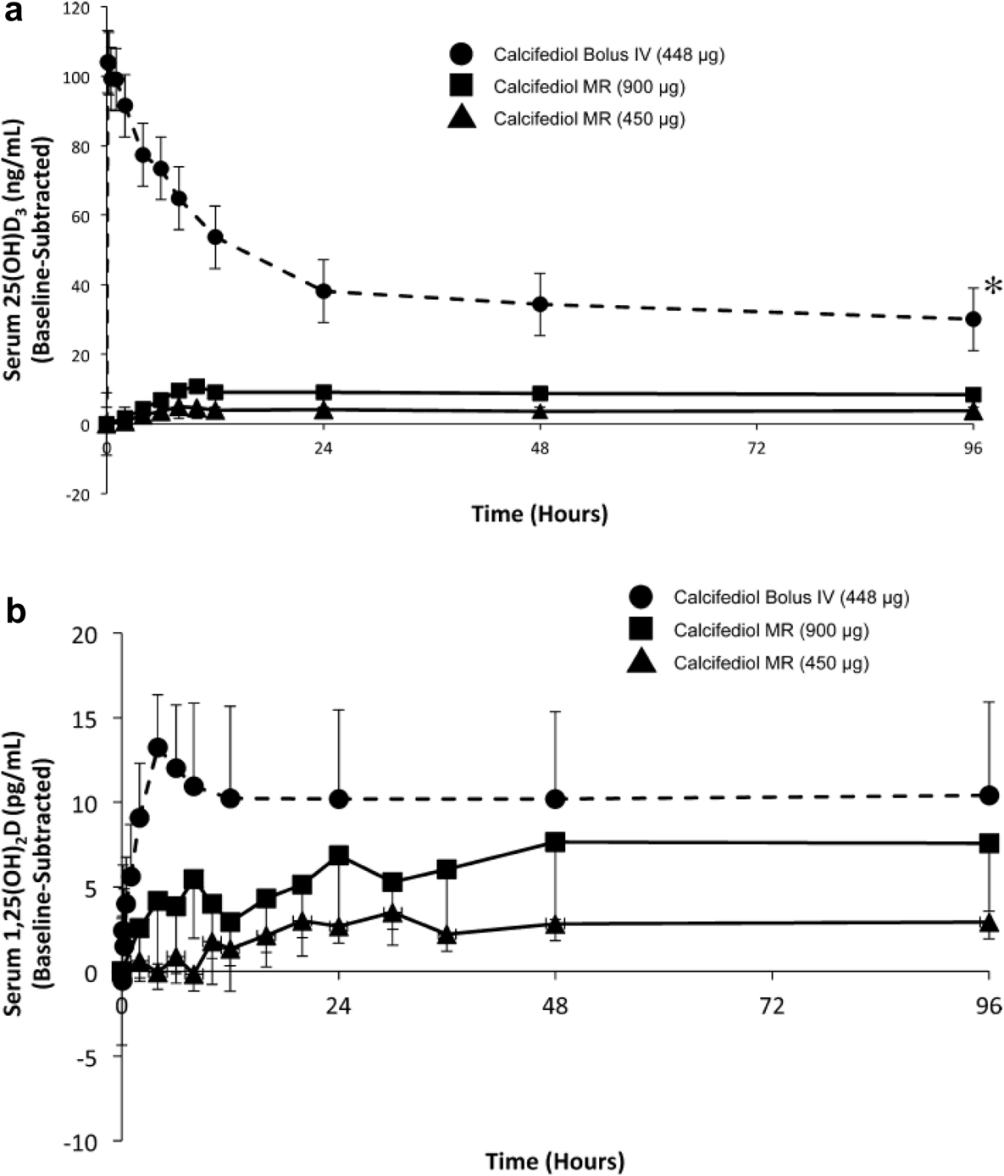
Effect of bolus i.v. or oral extended-release calcifediol administration on serum levels of calcifediol and 1,25-dihydroxyvitamin D in patients with stage 3 or 4 CKD, secondary hyperparathyroidism and vitamin D insufficiency. Patients received a single bolus i.v. injection of 448 mg calcifediol (solid circles) or single doses of oral extended-release calcifediol (450 mg—solid triangles; 900 mg—solid squares). Serum samples obtained at the indicated time points were analysed for **a** calcifediol (25(OH)D3) and **b** 1,25-dihydroxyvitamin D. Data are corrected for baseline values. Asterisk denotes significant differences at all time points post-treatment between i.v. and extended-release treatment groups (p < 0.05). MR: modified release formulation providing extended-release (From Petkovich et al. [42])

None of the agents in Fig. 1 has the ideal characteristics identified. In clinical practice, active vitamin D or an analogue is often prescribed to suppress parathyroid hormone in patients with secondary hyperparathyroidism.

In an observational cohort study conducted in 24 nephrology centres in Italy [25], prescription of active vitamin D or analogues was found to have progressively increased, starting from the early stages of CKD. However, guidelines from Kidney Disease Improving Global Outcomes (KDIGO) recommend that active vitamin D or analogues for secondary hyperparathyroidism treatment should be introduced only in late stages of CKD and end-stage renal disease in dialysis, and that their use should be reserved for patients with uncontrolled or rapidly increasing parathyroid hormone levels [26]. Active vitamin D and analogues are associated with a higher risk of hypercalcaemia [27]. Moreover, active vitamin D and analogues do not restore 25(OH)D levels and have the collateral effect of further reducing 25(OH)D levels by increasing FGF23. Because of this, active vitamin D and analogues must be combined with a nutritional vitamin D or a vitamin D prohormone (i.e., dual vitamin D therapy) [28].

Table 2 presents an overview of the 3 classes of vitamin D agents used for vitamin D repletion and modifying CKD-mineral and bone disorder parameters.

**Table 2:** Available treatment options for secondary hyperparathyroidism in patients with chronic kidney disease (Adapted from Cozzolino et al. [29])

Drug class	Drugs(s)	Current therapeutic uses	Mechanism and site(s) of metabolism/activation
Nutritional vitamin D	Cholecalciferol Ergocalciferol	Treatment/prevention of vitamin D deficiency; in combination therapies to treat bone conditions such as osteoporosis; maintenance of vitamin D levels, treatment of disease associated with vitamin D malabsorption	Physiological Activation needed (liver and kidney)
Non-1α-hydroxylated vitamin D prohormones	Immediate-release calcifediol	Prevention of vitamin D deficiency in renal failure and osteomalacia in adults, prevention of calcium disorders, treatment of rickets in children	Physiological activation needed (primarily kidney, some extra-renal tissues)
	Extended-release calcifediol	Treatment of secondary hyperparathyroidism in adults with stage 3 or 4 CKD and serum total 25-hydroxyvitamin D levels < 30 ng/mL	
Active vitamin D hormone	Calcitriol	Correction of calcium and phosphate metabolism abnormalities in adults with osteodystrophy; treatment of post-menopausal osteoporosis; treatment/prevention of secondary hyperparathyroidism^a^ in non-dialysis CKD and dialysis CKD	Pharmacological
1-α hydroxylated analogues	Paricalcitol Doxercalciferol Alfacalcidol	Correction of calcium and phosphate metabolism abnormalities in adults with osteodystrophy; treatment of post-menopausal osteoporosis; treatment/prevention of secondary hyperparathyroidism^a^ in non-dialysis CKD and dialysis	Hepatic activation needed for doxercalciferol and alfacalcidol

^a^Not all active vitamin D and analogues are indicated for secondary hyperparathyroidism

*CKD* chronic kidney disease

There is an urgent, unmet need for agents that can control parathyroid hormone levels and manage secondary hyperparathyroidism by optimizing serum 25(OH)D levels in pre-dialysis CKD patients who have vitamin D insufficiency or deficiency, without perturbing calcium and phosphate equilibrium or inducing excessive release of FGF23.

## Nutritional vitamin D

Evidence for supplementation with cholecalciferol or ergocalciferol in late-stage CKD is based on very limited clinical studies, both in quality and in statistical power, and have not shown consistent results with regard to parathyroid hormone suppression [30, 31].

## Prohormones

Vitamin D prohormones like 1α(OH)D, or 25(OH)D can be classified into those that require 1-α-hydroxylase activation, and those that are already hydroxylated in the 1-α-position. It is now known that extra-renal 1-α-hydroxylase can activate nutritional and prohormone vitamin D forms [32]; moreover, patients with pre-dialysis CKD have substantial renal 1-α-hydroxylase activity. Calcifediol (25-hydroxyvitamin D3) is a long-lasting form of prohormone (half-life approximately 25 days after repeated daily dosing in patients with stage 3 or 4 CKD) that provides substrate for 1-α-hydroxylation [21]. When administered in an immediate-release formulation, it increases 25(OH)D levels more rapidly and effectively than nutritional vitamin D, but is not effective at suppressing parathyroid hormone levels [33]. Immediate-release calcifediol reduces parathyroid hormone by clinically meaningful amounts (≥ 30%) only when administered at doses that both raise serum 25(OH)D to supra-physiological levels (> 100 ng/mL) and increase the risk of hypercalcaemia [29, 33–35]. This need for high doses of calcifediol to suppress parathyroid hormone may be due to an increase in FGF23 in response to the rapid rise in serum 25(OH)D levels obtained with immediate-release calcifediol.

## Active vitamin D

*Calcitriol* (1,25 dihydroxy vitamin D3) and other active (1α-hydroxylated) forms of vitamin D can effectively suppress parathyroid hormone levels in pre-dialysis patients with CKD; however, these agents are associated with an increased risk of hypercalcaemia and ectopic calcification, and do not replenish 25(OH)D substrate levels [36].

## Active vitamin D analogues

Like active forms of vitamin D, active analogues do not require activation by 1α-hydroxylase (CYP27B1), and therefore their activity is not subject to physiological regulation. They increase intestinal absorption of calcium and phosphorus, and their tolerability is limited by hypercalcaemia and/or hyperphosphataemia and the risk of vascular calcification [37–39]. As with active forms of vitamin D, these agents fail to correct circulating 25(OH)D levels that are important for extra-renal production of 1,25(OH)2D [40, 41], and therefore must be administered together with nutritional vitamin D or prohormones. Finally, these active analogues can trigger the feedback mechanism of FGF23-induced vitamin D degradation, further reducing vitamin D levels. Because of this, and in the absence of data supporting an improvement in hard clinical outcomes, they are not recommended for routine use in patients with pre-dialysis CKD unless they have severe and progressive hyperparathyroidism [26].

## Extended-release calcifediol

An extended-release formulation of calcifediol has been developed to treat secondary hyperparathyroidism in non-dialysis patients with CKD. Rayaldee^®^ (OPKO Health/Vifor Fresenius Medical Care Renal Pharma) is an orally administered, extended-release formulation of 25(OH)D3 that is approved in the US, Canada and Europe for treating adults with stage 3–4 CKD and vitamin D insufficiency [21, 22]. Rayaldee^®^ is formulated in capsules containing 30 μg of calcifediol monohydrate in a lipophilic excipient mixture (mineral oil, mono- and diglycerides, paraffin, hydroxypropyl methylcellulose, lauroyl polyoxylglycerides, dehydrated alcohol and butylated hydroxytoluene) [21], which provides extended release over a 12-h period [42]. Steady-state levels of 25(OH)D are achieved after approximately 3 months of treatment [43].

After administration of Rayaldee^®^, 25(OH)D3 is extensively (> 98%) bound to plasma proteins. The mean apparent volume of distribution is 8.8 L in healthy subjects following a single oral dose of Rayaldee^®^, and 30.1 L in patients with stage 3 or 4 chronic kidney disease following repeated dosing [21].

## Pharmacology

The extended-release strategy is designed to gradually increase serum total 25(OH)D concentrations to targeted levels, while avoiding upregulation of FGF23 and vitamin D catabolism through CYP24A1. Moreover, activation of this prohormone is tightly controlled at renal and extra-renal levels by the requirement for CYP27B1 1α-hydroxylase activation to 1,25(OH)2D, thereby reducing the risk of hypercalcaemia and ectopic calcification [42].

In patients with stage 3/4 CKD, single-dose administration of extended-release calcifediol did not cause a rapid rise in 25(OH)D or trigger catabolism. In a proof-of-concept study, 29 patients with stage 3/4 CKD, secondary hyperparathyroidism and vitamin D insufficiency (serum total 25(OH)D < 30 ng/mL) were randomised to receive either a single bolus i.v. injection of calcifediol (448 µg) or a single oral dose of extended-release calcifediol (either 450 µg or 900 µg) [42]. The rapid rise in serum 25(OH)D after the i.v. bolus (Fig. 1a) resulted in a rapid rise in activated 1,25(OH)2D that was not observed with either of the oral doses administered (Fig. 1b).

As shown in Fig. 1, only a small fraction of the circulating 25(OH)D (Panel A) is transformed into activated 1,25(OH)D (Panel B), i.e., less than 0.1% on average [44]. When steady plasma levels are achieved after 48 h, the estimated conversion rates after the administration of 450 and 900 µg of Rayaldee^®^ are 0.075 and 0.094%, respectively, which is approximately 3-fold higher compared to 0.03% observed after administration of an i.v. bolus of 448 µg of 25(OH)D.

Thus, the more gradual increase in plasma 25(OH)D levels obtained with Rayaldee^®^ translates into an optimal conversion rate, most likely because the subsequent slower increase in activated 1,25(OH)D reduces its own catabolism by CYP24A1 [42]. Interestingly, the 25(OH)D conversion rate 48 h after administration of 900 µg of Rayaldee^®^ appears to be higher in patients with CKD compared to healthy subjects (0.094 vs. 0.053%, respectively) [Unpublished data on file]. However, a relationship between the rate of 25(OH)D conversion and its clinical efficacy in CKD remains to be established.

Pharmacokinetic data on extended-release calcifediol provide the basis to explore the relationships existing between circulating 25(OH)D levels and biomarkers of CKD. Parathyroid hormone levels decreased significantly with extended-release calcifediol 900 mg, compared to the i.v. calcifediol bolus.

**Fig. 2:**
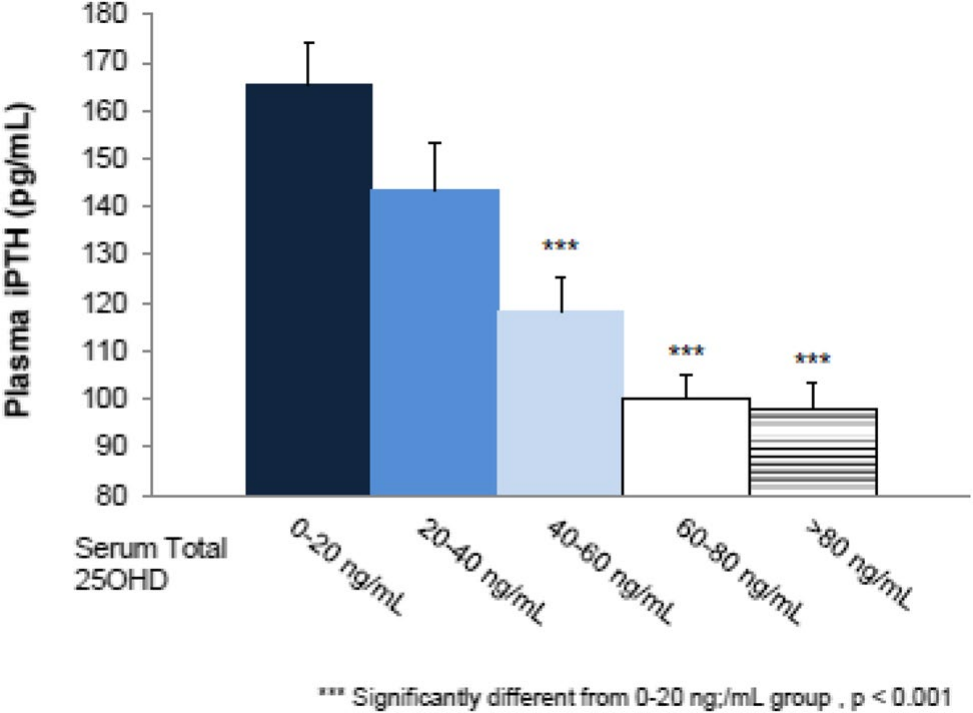
Mean (± SE) plasma intact parathyroid hormone versus mean serum total 25-hydroxyvitamin D (ng/mL) during the efficacy assessment period in the per protocol population (Sprague et al. [24])

**Fig. 3:**
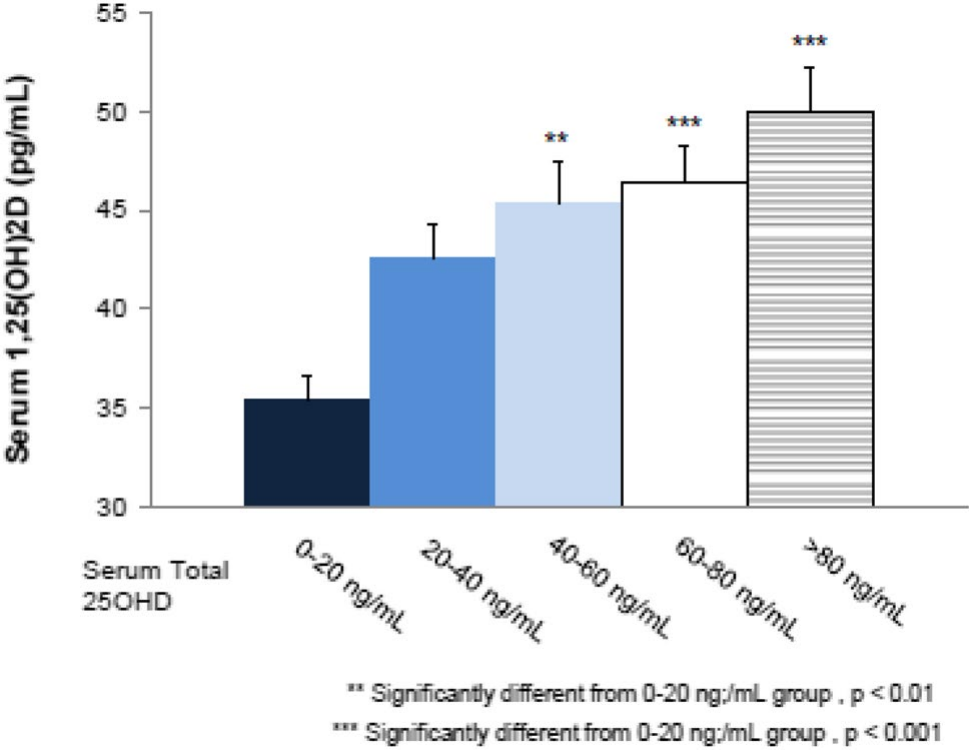
Mean (± SE) serum total 1,25-dihydroxyvitamin D (pg/mL) versus serum total 25-hydroxyvitamin D (ng/mL) during the efficacy assessment period in the per protocol population (Sprague et al. [24])

In a secondary analysis of pooled data from the 2 most recent RCTs conducted with extended-release calcifediol in patients with stage 3 or 4 CKD [19], the mean plasma concentration of intact parathyroid hormone at the end of treatment was inversely proportional to the mean serum 25(OH)D concentration (Fig. 2). In contrast, mean serum total 1,25(OH)_2_D concentration was directly proportional to the mean serum 25(OH)D concentration (Fig. 3). These findings support the conclusion that Rayaldee^®^ lowers elevated plasma intact parathyroid hormone by raising serum total 25(OH)D, and that the mechanism for plasma intact parathyroid hormone suppression is elevation of serum total 1,25(OH)2D.

## Clinical studies

**Fig. 4:**
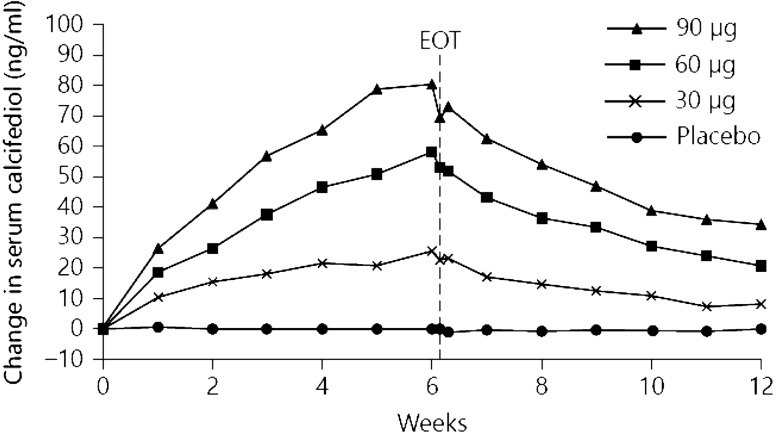
Changes from baseline in serum 25(OH)D concentrations during the 6-week treatment period according to the extended-release calcifediol dose administered (30, 60 or 90 μg/day). EOT, end of treatment (From Sprague et al. [17])

Sprague et al. conducted a 12-week randomized, double-blind, placebo-controlled trial that evaluated the efficacy of extended-release calcifediol in 78 patients with CKD, parathyroid hormone > 70 pg/mL and serum total 25(OH)D < 30 ng/mL [17]. Patients were randomised to receive daily oral extended-release calcifediol (30, 60 or 90 μg), or placebo. Serum 25(OH)D levels increased in proportion to dosage (Fig. 4). At end of treatment after 6 weeks, serum 25(OH)D levels had normalised (≥ 30 ng/mL) in 90% of patients treated with extended-release calcifediol vs 3% with placebo (p < 0.0001), and mean serum 25(OH)D among all extended-release calcifediol-treated patients was 64.4 ± 24.9 ng/mL vs 18.5 ± 5.3 with placebo (p < 0.0001). extended-release calcifediol was well-tolerated and there were no clinically significant safety concerns.

**Fig. 5:**
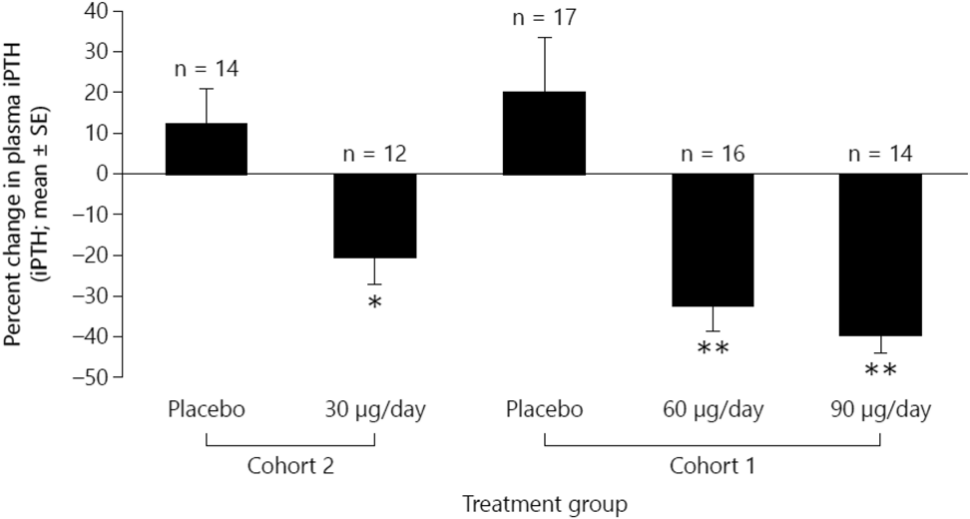
Percent changes from baseline in plasma intact parathyroid hormone at the end of the 6-week treatment period according to the administered extended-release calcifediol dose (30, 60 or 90 μg/day). *Significantly different from placebo, p < 0.05; **Significantly different from placebo, p < 0.001 (From Sprague et al. [17])

The percent change from baseline in parathyroid hormone with extended-release calcifediol increased with increasing extended-release calcifediol dosage administered (Fig. 5).

**Fig. 6:**
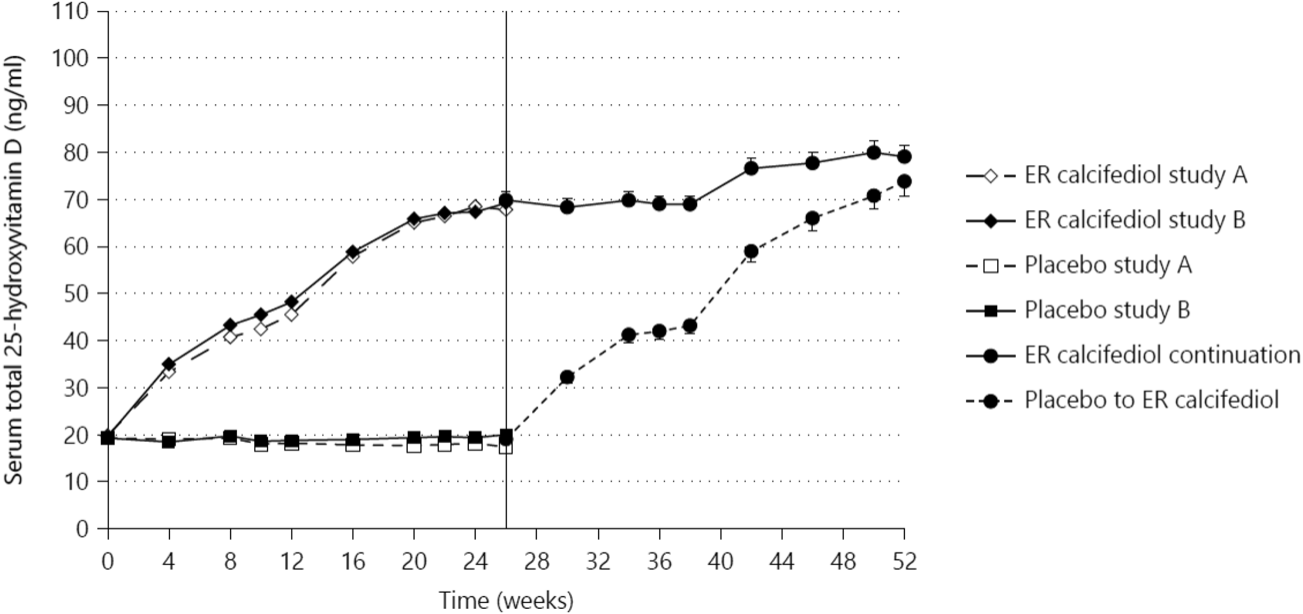
Mean (SE) change over time in serum total 25(OH)D in the combined per protocol population. Data points from 0 to 26 weeks represent mean values for individual time points from placebo-controlled studies A and B. Error bars in this portion of the figure are omitted for clarity. Data points from 26 to 52 weeks represent mean ± SE values for data from the open-label extension study. SE values for 0–26 weeks were of similar magnitude to those in the open-label extension (From Sprague et al. [44])

The efficacy and safety of extended-release calcifediol were evaluated further in two identical 26-week randomised, placebo-controlled, double-blind trials in patients with stage 3/4 CKD, secondary hyperparathyroidism, and vitamin D insufficiency (serum total 25(OH)D 10–30 ng/mL) [43]. Patients were randomized (2:1) to receive extended-release calcifediol once daily (30 µg for 12 weeks, followed by either 30 or 60 µg for 14 weeks), or placebo. At week 13, the extended-release calcifediol dose was increased if serum parathyroid hormone was > 70 pg/mL, serum 25(OH)D was < 65 ng/mL, and serum calcium level remained < 9.8 mg/dL. In a 26-week, open-label extension of these two studies, 298 patients continued receiving their extended-release calcifediol dosing level or crossed over from placebo to extended-release calcifediol (Fig. 6).

Extended-release calcifediol normalized serum total 25(OH) D levels in > 95% of the per protocol population and was well-tolerated, without clinically relevant effects on serum calcium, phosphorus, or FGF23 levels. The lack of effect on FGF23 suggests that extended-release calcifediol does not activate the vitamin D catabolic feedback mechanism involving 24-hydroxylase [19].

Oversuppression of parathyroid hormone is a concern in patients with stage 3–4 CKD treated with calcitriol or active analogues because low parathyroid hormone concentration is associated with adynamic bone disease and hypercalcaemia [26, 36]. Randomized controlled trials have shown that extended-release calcifediol gradually but effectively reduces parathyroid hormone levels without causing oversuppression.

Post hoc analysis of this data examined the impact of baseline parathyroid hormone levels on end-of-treatment parathyroid hormone levels [45]. Extended-release calcifediol and placebo had similar, minor effects on mean serum calcium and phosphorous. Extended-release calcifediol increased serum 25(OH)D and 1,25(OH)_2_D significantly and to comparable levels regardless of baseline intact parathyroid hormone tertile. However, decreases in mean intact parathyroid hormone with extended-release calcifediol differed between baseline intact parathyroid hormone tertiles and were directly proportional to baseline levels. Oversuppression of intact parathyroid hormone was not observed. The mean absolute intact parathyroid hormone reductions that were proportional to baseline intact parathyroid hormone levels is consistent with a mechanism of action involving physiological regulation of intact parathyroid hormone modulated by secondary hyperparathyroidism severity.

Strugnell et al. [19], conducted a post hoc analysis of aggregated data from two phase 3 studies by Sprague et al. [17, 43] to examine possible relationships between post-treatment 25(OH)D quintile and associated changes in factors relevant to CKD-mineral and bone disorder, including plasma intact parathyroid hormone, serum bone turnover markers, calcium, phosphorus, intact FGF23 and vitamin D metabolites, estimated glomerular filtration rate, and urine calcium/creatinine ratio.

**Fig. 7:**
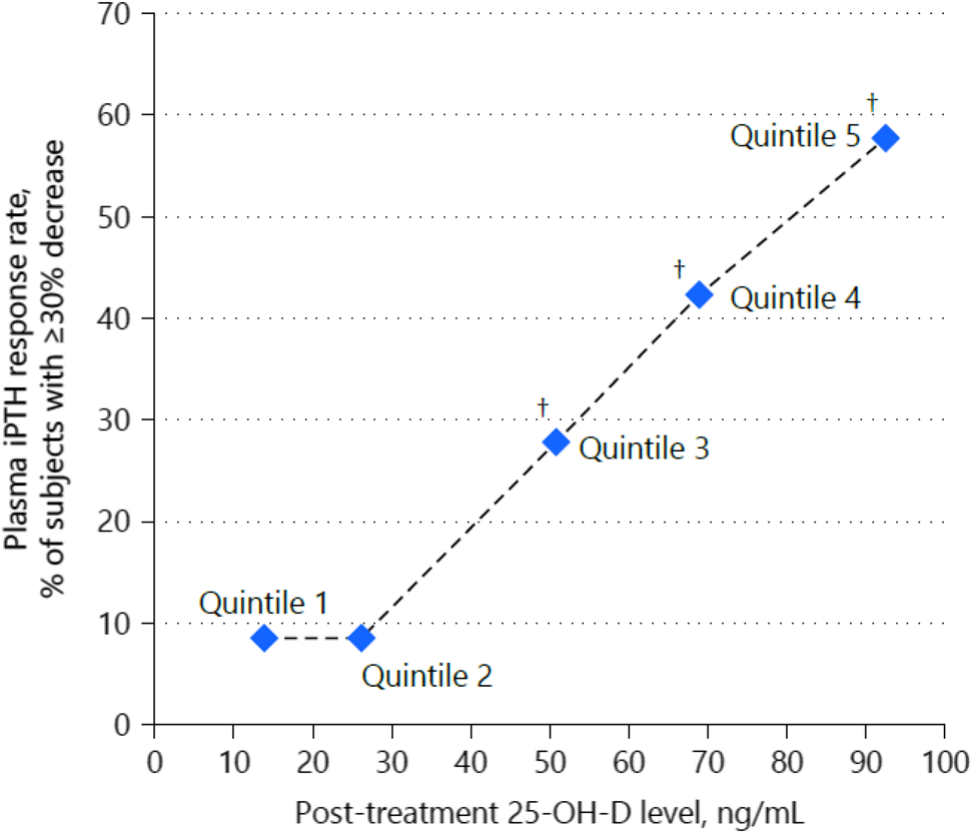
Analysis of plasma intact parathyroid hormone response rates by posttreatment 25(OH)D Quintile. The proportion of per protocol subjects achieving an intact parathyroid hormone response, defined as a mean decrease of ≥ 30% in plasma intact parathyroid hormone from pre-treatment baseline, was analysed as a function of mean posttreatment serum total 25(OH)D quintile (from Strugnell et al. [19]). ^†^Significantly different from Quintile 1, p < 0.05; *iPTH* intact parathyroid hormone

Progressive increases in serum 1,25-dihydroxyvitamin D and reductions in plasma intact parathyroid hormone and serum bone turnover markers were observed as mean posttreatment serum 25(OH)D rose from 13.9 ng/mL (in Quintile 1) to 92.5 ng/mL (in Quintile 5), regardless of CKD stage. Mean serum calcium, phosphorus and FGF23 levels, estimated glomerular filtration rate and urine calcium/creatinine ratio (collectively “safety parameters”) did not change significantly from Quintile 1. Suppression of intact parathyroid hormone and bone turnover markers was not observed until serum 25(OH)D rose to at least 50.8 ng/mL (Quintile 3) (Fig. 7). This has been interpreted as an indication that current targets for vitamin D repletion therapy in CKD may be too low [19], because the mean serum 25(OH)D of 92.5 ng/mL in the 5^th^ quintile was not associated with undesirable changes in safety parameters, but did result in a progressive increase in the percentage of patients with parathyroid hormone reductions ≥ 30%.

Thus, meaningful reductions in parathyroid hormone require serum 25(OH)D levels above the guideline-recommended targets [20]. The results also suggest that higher serum 25(OH)D levels can be achieved in patients with stage 3/4 CKD without sacrificing safety. These findings are consistent with the results of a large cross-sectional analysis of 14,289 unselected patients with CKD stage 1–5 that revealed a progressive lowering of parathyroid hormone up to 25(OH)D levels of 42–48 ng/mL, with a somewhat decreasing response above that level [18]. Also in that study, there was no association between higher 25(OH)D levels and hyperphosphataemia or hypercalcaemia.

A recent real-world study conducted on clinical records from 18 Nephrology clinics in the US compared the effects of nutritional vitamin D (n = 147), extended-release calcifediol (n = 174), and active vitamin D agents (n = 55) on levels of 25(OH)D, parathyroid hormone, calcium, and phosphorus levels after ≥ 20 weeks of follow-up in adults with stage 3 or 4 CKD. The clinical effectiveness and safety of extended-release calcifediol in the real-world setting was consistent with what has been reported in randomised clinical trials. Only extended-release calcifediol was associated with a statistically significant decrease in parathyroid hormone (− 34 ng/mL; p < 0.001). Extended-release calcifediol did not have a statistically significant effect on serum calcium or phosphate levels [46].

## Position of extended-release calcifediol in current therapy for secondary hyperparathyroidism

Treatment of secondary hyperparathyroidism in patients with CKD stages 3 or 4 and vitamin D deficiency should aim to control parathyroid hormone levels early in the disease course, while parathyroid cells are still responsive to physiological 1,25(OH)2D signalling. Although there is no consensus on the optimal serum 25(OH)D concentration or parathyroid hormone targets in patients with secondary hyperparathyroidism, recent evidence suggests that 25(OH)D levels > 50 ng/mL are required to effectively reduce parathyroid hormone levels [18].

Guidelines recommend nutritional vitamin D supplements for treating secondary hyperparathyroidism in patients with non-dialysis CKD, and suggest that active vitamin D (calcitriol) and analogues should be reserved for patients with advanced CKD, uncontrolled parathyroid hormone levels that are rising quickly, and for patients on dialysis [20]. However, nutritional forms of vitamin D and immediate release formulations of calcifediol have only modest effects on parathyroid hormone levels and are associated with increased risk for hypercalcaemia, especially when administered at high doses; moreover, these agents appear to trigger catabolism of vitamin D sterols.

Calcitriol and active vitamin D analogues are effective for lowering parathyroid hormone levels but are associated with even higher risks of hypercalcaemia. At the same time, they must be administered with a nutritional vitamin D agent (dual therapy) because they do not replenish 24(OH)D substrate to support pleiotropic (autocrine/paracrine) functions.

Several characteristics of extended-release calcifediol support its use in the intermediate stages of CKD in patients with secondary hyperparathyroidism and vitamin D insufficiency. Extended-release calcifediol reduced parathyroid hormone levels to an extent comparable to what can be achieved with the combination of active vitamin D analogues combined with natural vitamin D agents. In clinical trials, monotherapy with extended-release calcifediol in patients with stage 3 or 4 CKD achieves dose-dependent increases of 25(OH)D and physiological increases in 1,25(OH)2D, accompanied by sustained reduction of parathyroid hormone and little or no impact on mineral balance or FGF23 levels. Meanwhile, emerging data from real-world clinical experience with extended-release calcifediol for patients with stage 3 or 4 CKD suggest that it is comparable to active vitamin D analogues for controlling parathyroid hormone levels, but has the added benefit of replenishing 25(OH)D [46].

**Fig. 8:**
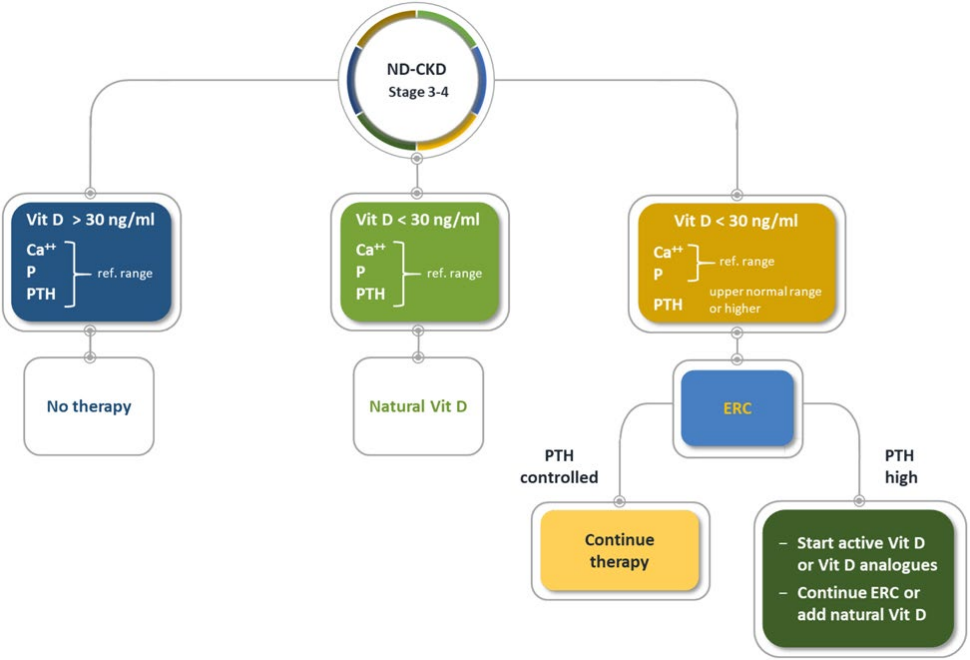
Flowchart for managing patients with nondialysis-CKD stage 3–4. *ERC* extended-release calcifediol, *parathyroid hormone* parathyroid hormone, *Vit D* vitamin D

**Fig. 9:**
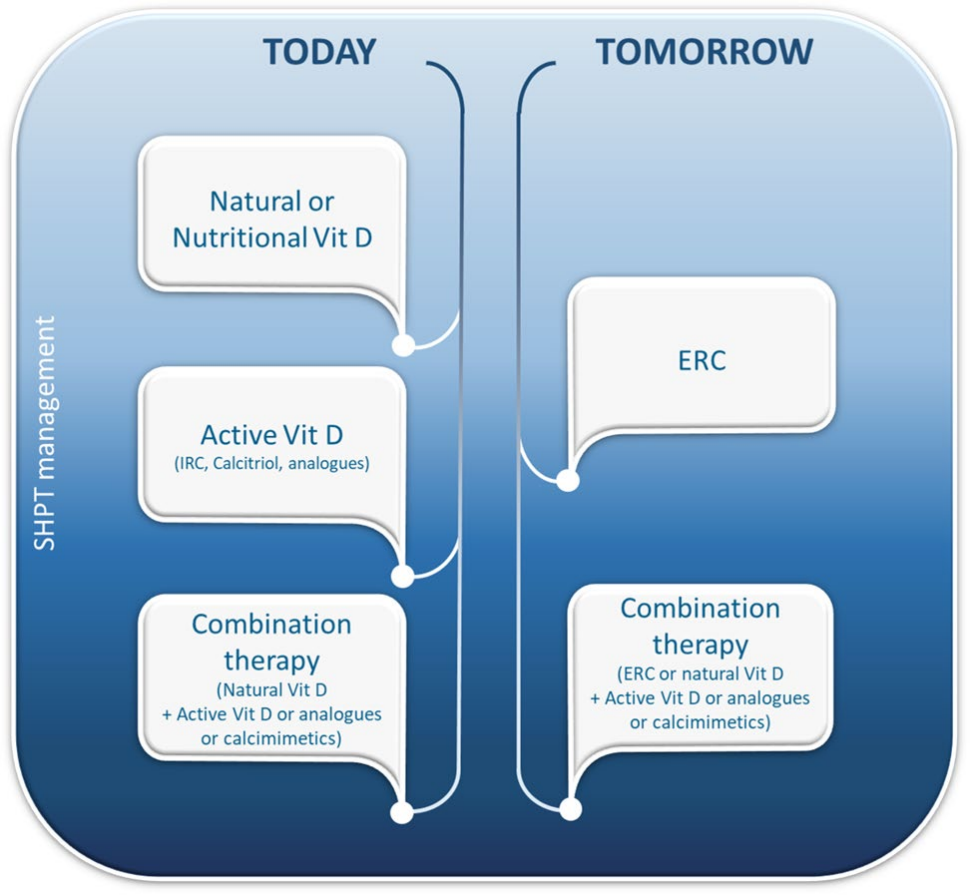
Current and future management of secondary hyperparathyroidism in patients with CKD and hypovitaminosis D. *Vit D* vitamin D *IRC* immediate-release calciferol, *ERC* extended-release calciferol, *secondary hyperparathyroidism* secondary hyperparathyroidism

In Fig. 8, we propose a flowchart for treating secondary hyperparathyroidism in patients with CKD stage 3–4. In patients with serum 25(OH)D levels >= 30 ng/mL and serum Ca, P, and parathyroid hormone in the normal range, there is no need to start therapy for secondary hyperparathyroidism. In patients with 25(OH)D < 30 ng/mL and serum Ca, P, and parathyroid hormone in the normal range, we suggest using nutritional vitamin D, such as cholecalciferol. In patients with 25(OH)D < 30 ng/mL and parathyroid hormone levels in the upper normal range or higher, we suggest administering ERC, which should be continued if secondary hyperparathyroidism is controlled; if serum parathyroid hormone levels remain persistently elevated, we suggest continuing extended-release calcifediol therapy or replacing it with a nutritional vitamin D and combining this with an active vitamin D or vitamin D analogue (Fig. 9).

## Conclusion

Treatment for patients with intermediate-stage non-dialysis CKD and vitamin D deficiency should aim to replenish 25(OH)D levels with a nutritional form of vitamin D. If secondary hyperparathyroidism is present, treatment should aim to control parathyroid hormone levels early in the disease course. This may require targeting serum 25(OH)D levels > 50 ng/mL. Nutritional forms of vitamin D and immediate release formulations of calcifediol have only modest effects on parathyroid hormone levels. In this setting, monotherapy with extended-release calcifediol achieves dose-dependent increases of 25(OH)D and physiological increases in 1,25(OH)2D, with sustained reduction of parathyroid hormone levels, comparable to what can be achieved with active vitamin D analogues; however, extended-release calcifediol does so with less risk of hypercalcaemia and has the added benefit of also replenishing 25(OH)D.

## Data Availability

Not applicable.
